# Dog Ecology and Demographics in Several Areas in the Philippines and Its Application to Anti-Rabies Vaccination Programs

**DOI:** 10.3390/ani12010105

**Published:** 2022-01-02

**Authors:** Amit Chaudhari, Tamara Kartal, George Brill, Kazami Joanne Amano, Maria Glofezita Lagayan, Daphne Jorca

**Affiliations:** 1 Humane Society International, 2100 L St., NW, Washington, DC 20037, USA; tamarakartal1@gmail.com (T.K.); gtbrill26@gmail.com (G.B.); 2 Independent Consultant, Davao City 8000, Philippines; kazamijoanneamano@gmail.com; 3 Bureau of Animal Industry (BAI), Department of Agriculture, Visayas Avenue, Diliman, Quezon City 1101, Philippines; opulenciajoy@gmail.com (M.G.L.); daphne.jorca@bai.gov.ph (D.J.)

**Keywords:** dog population, household survey, dog, dogs per 1000 humans, dog density, anti-rabies vaccination, mobile phone application, mass vaccination, rabies

## Abstract

**Simple Summary:**

Dog population estimates are necessary to design effective rabies and dog population control programs. Dog population sizes vary drastically from country to country and vary within a country based on human tolerance, pet ownership practices, culture, religion, and several other factors. Human density, level of urbanisation and human settlement types (urban, semi-urban and rural) also play a role in the size of the dog population. Humane dog management programs have shown that dog density per km street length is one measure to monitor the program’s impact. However, we argue here that efficient sterilisation and vaccination program planning also requires an estimate of the total dog population. In the Philippines, we have conducted owned dog population surveys (household surveys and dog demographic surveys), which have proven to be very effective in planning high-volume vaccination programs. Following the implementation of the dog surveys and the subsequent understanding by local officials that actual dog populations were far higher than originally assumed, a higher rabies vaccination coverage was achieved in two target cities due to a correction in the number of vaccines doses needed.

**Abstract:**

Understanding dog population dynamics plays a vital role in planning both rabies and dog management interventions. Establishing a human to dog ratio and an understanding how the urban/rural nature of the community might affect the overall dog population estimate provides an easy-to-use reference to estimate approximate dog populations in a range of communities. A total of 10,664 households were interviewed in 10 locations in the Philippines (2017 and 2018) to understand the dog population variations among the urban, semi-urban and rural areas. Epicollect5 and OSM tracker applications were used to conduct household interviews using a predesigned fixed set of questions. All answers were recorded directly using mobile phone applications. The survey results showed that for every 1000 humans, there are 256.3 dogs in rural areas, 213.8 dogs in semi-rural areas, 208.7 dogs in urban areas and 170.0 dogs on small islands of the Philippines. We estimate a total dog population in the Philippines of 23.29 million dogs (CI 95%, 22.51–24.07 million). Based on the survey findings from Quezon City and Cebu City, targets, resources allocations and vaccination approach were adjusted for the anti-rabies vaccination program at two locations in 2018, which lead to a 3- to 4-fold increase in the total number of dogs vaccinated in each city compared to previous years.

## 1. Introduction

Dogs have evolved to be with humans or close to human settlements for survival [1]. Dogs are usually very dependent on human food provision and the relative dog population (e.g., dogs per 1000 people) fluctuates according to the level of human tolerance [2]. In many countries (mostly high income), the relationship between humans and dogs has reached the stage where dogs are part of the family and are mostly very well taken care of. However, there are still large populations of roaming dogs living in harsh conditions on the streets in Asia, Africa, and Latin America. It is usually difficult to distinguish the ownership status of free-roaming dogs; therefore, it is not unusual for owned but partly roaming dogs to be identified as unowned dogs. There is growing evidence that these free-roaming dogs are mostly owned not dependent on garbage, but rather dependent on the direct provision of food by humans [3,4,5].

In the last ten to twenty years, the number of papers reporting dog populations (both owned and roaming on the streets) has increased significantly [6,7,8]. These dog census projects have often been conducted to provide estimates of the proportion of dogs that need to be vaccinated against rabies (it is usually assumed that one has to vaccinate 70% of a dog population to eliminate the transmission of rabies among dogs and hence to people).

To plan an effective rabies management program, it is vital to have a realistic estimate of the number of dogs present in specific areas of interest. Unfortunately, estimating actual population size with high accuracy can be a resource-intensive and complicated process. For that reason, it is often more feasible to approximate dog population sizes using simpler kinds of metrics. One option is to conduct counts of roaming dogs along transects and use the resulting data to calculate an index of density describing the number of dogs seen per km of the transect. Although this index does not give true population size, it does provide a valuable indicator of the relative density of dogs in different areas or during different periods. Another approach is to use a proxy variable—usually human density, which is typically known—as an indicator of likely dog density. This is often expressed as the number of dogs present per 1000 people. Data to generate this metric can be obtained with transect surveys, household questionnaires or a combination of these methods. Each of these methods has its own strengths and weaknesses and, furthermore, they tend to sample overlapping, but not equivalent segments of the total dog population. If we know what knowledge exists among the people and what practices they follow, it will help planning a more effective program. It also provides an opportunity to program implementor to identify hurdles and find realistic solutions.

Several methods to estimate dog population are available, often consisting of a combination of questionnaire surveys and street counts, depending on the dog demographics in a community [9]. Humane Society International (HSI) has conducted numerous dog surveys in Asia and Africa has reported the relative dog population in particular communities, i.e., the number of dogs per 100 or 1000 humans. HSI has also conducted numerous KAP (Knowledge, Attitude and Practice) surveys to understand dog ownership practices and to estimate sterilisation and vaccination program costs. One very simple method of estimating the impact of a sterilisation program on street dog population is the development of one or more index survey routes where the number of dogs observed along the route at set times and times of year are counted and plotted on a graph showing the changes in observed dog numbers and sterilisation status [10]. Taking only free-roaming dogs count and measuring dog density per km is not sufficient for owned roaming dog populations, as the roaming dog density is under the direct control of dog owners.

Dogs on the street are at high risk of contracting disease from other dogs as well as creating a risk to the community. Zoonotic diseases, including rabies, are a serious concern for many governments [11]. Free-roaming dogs and rabies transmission are closely linked in many low-income countries and large unmanaged dog populations are a particularly daunting challenge for rabies control [12,13]. Understanding the demography of domestic dogs is essential when planning a dog population management and rabies control program [14,15].

The lack of dog population estimates has led to ineffective rabies vaccination programs [16]. The US Centers for Disease Control and Prevention (CDC) developed a tool for vaccination campaign planning that requires dog demographic data to develop an appropriate vaccination program, including data on free-roaming owned, free-roaming unowned and owned confined dogs [17]. Most dogs in the developing world are short-lived and the high turnover rapidly reduces the level of vaccination coverage in a dog population [18]. Several anti-rabies vaccination programs in Asia and Africa have not achieved the required 70% vaccination coverage, the target level of vaccination that is projected to break rabies transmission in a dog population for twelve months [16].

In the Philippines, vaccinating dogs against rabies and registering owned dogs with the local government authority is mandatory. Keeping a dog on a leash when in public places is also mandatory by law (Republic Act No. 9482 “Anti-rabies Act of 2007”—a system for the control, prevention of the spread and eventual eradication of human and animal Rabies shall be provided and the need for responsible pet ownership established) [19]. Nevertheless, free-roaming dogs on the street are common and dog-mediated rabies is prevalent across the Philippines [20]. The National Rabies Prevention and Control Manual of Procedures in the Philippines provides guidelines on the estimation of the dog population. The most common estimate when planning rabies control projects has been that there is 1 dog per 10 humans [20]. 

Rabies is endemic in the Philippines and remains a major public health concern. It has a fatality rate of almost 100% and at least one-third of these deaths occur in children aged 15 years old and below. Nationally, the number of animal bite cases in the country increased by 462%, from 2009 (206,253 bite cases) to 2018 (1,159,711 bite cases) [20]. The confirmed number of positive human rabies cases increased by 13.5% between 2009 and 2018, from the 243 cases reported in 2009 to 276 in 2018 [20]. 

## 2. Materials and Methods

In preparation for a new strategy of anti-rabies vaccination programs, the Department of Agriculture Regional Field Offices (DARFO) of the Bureau of Animal Industry (BAI) and Humane Society International conducted cross sectional surveys to generate dog population estimates and densities across urban and rural areas. Ten different urban, semi-urban and rural locations were surveyed either based on local governments request or as a part of an evidence-based rabies control program strategy that BAI and HSI agreed to develop. These surveys were conducted at different times and by different team combinations between 2017–2018. A standard method and established protocol were followed during each location’s survey. A short questionnaire to record dog demographics targeting randomly selected households in all locations was developed. However, in Quezon City, a longer questionnaire was added to explore Knowledge, Attitudes and Practices (KAP) in the selected barangays (A small territorial and administrative district forming the most local level of government).

### 2.1. Phone Based Application and Survey Questionnaire

Two different smartphone applications were used for household surveys: Open Street Map (OSM) Tracker with a layout specifically designed for a shorter version of the questionnaire and Epicollect5 (https://five.epicollect.net/, accessed on 27 December 2021) to conduct the longer version. The shorter version was designed to reach a large number of households and was used at all the survey locations. The longer questionnaire was used only in Quezon City in addition to the shorter version. 

**Figure 1:**
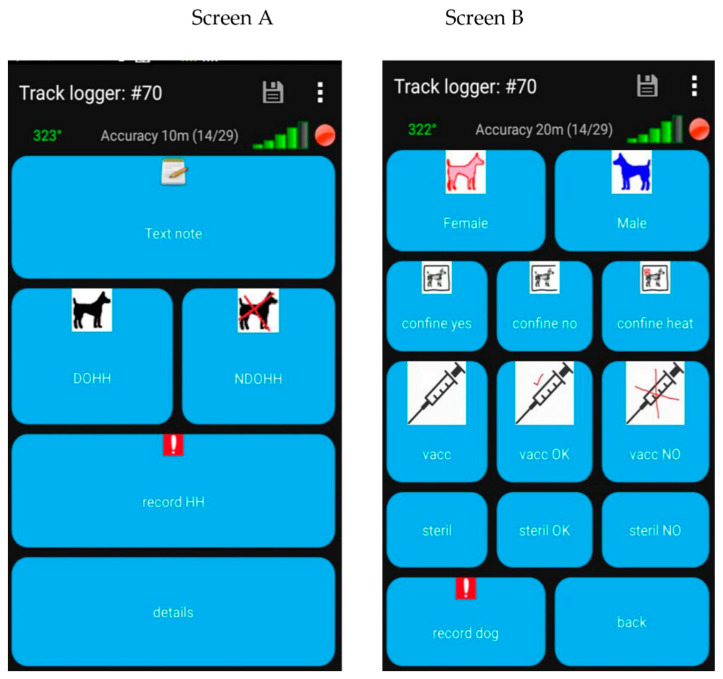
Screenshots of OSM Tracker application with modified layout showing icons-based screen used in shorter version to conduct household surveys at all the locations.

The short version included an icon-based set of questions for dog-owning households (DOHH) and no dog-owning households (NDOHH). The surveyor selects an icon on the OSM Tracker display to record the answers (Figure 1). Each surveyed household was asked whether or not they owned one or more dogs and in the event of a dog-owning household, the surveyor further asked the sex of the dog, confinement practices followed by the owner, sterilisation and vaccination status and willingness to vaccinate and sterilize. The data in the form of GPX files were uploaded to a specifically designed Access database to produce results from uploaded GPX files. Screen A (Figure 1) was the main screen used to record if participant’s house is DOHH or NDOHH, whereas screen B (Figure 1) appeared upon selecting the ‘details’ icon on the main screen and was used to record each dog’s details in the case of DOHH.

Each household approached for the survey was asked the questions below in the shorter version; the questions were in the form of icons on the OSM Tracker. The questions indicated by the icons on the screens are listed below.

**Screen A:** DOHH—Dog owning household, NDOH—Non dog owning household; **Screen B:** In the case of DOHH, further, we asked below detail for each owned dog.

- Gender of dog—Female, Male
- Confinement practices followed by owner—Confined all the time (confine yes), not confined at all the time (confine no) and Female dog confined during the heat (confine heat)
- If the dog was vaccinated against rabies in the last one year and, in case of un-vaccinated dog, surveyor to further asked owner’s willingness for vaccination if it were to be provided free—Vaccinated in the last one year (vacc), willing to vaccinate (vacc OK) and not willing to vaccinate (vacc NO)
- If the dog was sterilized or not and in case of an unsterilized dog, owner’s willingness to sterilize if it were to be provided free—Sterilized (steril), willing to sterilize (steril OK) and not willing to sterilize (steril NO)

It was considered culturally insensitive to ask if the female dog was tethered during the heat (confine heat) and so it was not asked at any surveyed locations.

The longer version of the questionnaire (conducted only in Quezon City) collected more detailed information on household demographics, knowledge, attitudes regarding dogs, rabies prevention and dog bite wound care knowledge should a household member be bitten and knowledge concerning the rabies program and human attitudes to rabies and dog management practices in Quezon City. The set of questions was created in the Epicollect5, which allows questions not applicable to the participant to be skipped. For example, if a household does not own a dog, upon selecting NDOHH, the questionnaire skipped the questions related to dog details. 

To maintain consistency in data collection at each location, surveyors were given two days of indoor (theoretical) and outdoor in situ (practical) survey protocol training before starting household survey. Each survey team consisted of two individuals, either two government employees or one government employee and one Humane Society International staff. For each location, several teams were trained and employed for household surveys.

### 2.2. Survey Design and Household Selection

The local government provided digital map files for each survey location to identify and locate each barangay via Google Maps. Using the most recent census data, barangays were stratified into human density categories (rural and urban) and an Excel sheet was used to draw a random sample of the barangays for survey by generating unique random numbers in excel using the RAND function. Each randomly selected barangay was marked on Google Maps and shared with the survey team. Table 1 provides a listing location and the sample size for each survey. For example, Cebu City areas were divided into rural and urban areas based on human population density. Rural areas were defined as those with a human population below 5000 humans per square kilometer. Urban areas were defined as those with more than 9000 humans per square kilometer. Thirty rural barangays and fifty urban barangays were classified. The barangays were distributed across the North and South regions, with 46 barangays located in the north and 34 barangays in the south of Cebu City. This gave us four identified categories: North Rural (16 barangays), North Urban (30 barangays), South Rural (14 barangays) and South Urban (20 barangays). Using the free online sample size calculator, Raosoft^®^, it was determined that 2020 households would need to be surveyed to achieve a confidence level of 95% for the owned dog population survey. The final sample included 4 North Rural barangays, 5 North Urban barangays, 3 South Rural barangays and 6 South Urban barangays that were randomly selected using Microsoft Excel. The household sample size to be surveyed per barangay varied from 40 to 240. This was dependent on the barangay’s population density and the number and spatial distribution of households.

Survey teams received a map/area boundary in Google Maps with marker pins indicating where they would conduct the surveys. Households were selected in a stratified random sampling method around the pins. A target number of households were interviewed, walking zigzag in small rectangle or square area around the marker pin, making sure not to reach closer to another marker pin and interviewing every 5th or 10th household either on the right or left side only. 

Additionally, barangays in Quezon City, where we conducted the KAP survey were selected based on the available bite and rabies statistics for both humans and animals (provided by the City Veterinary Offices). Priority was given to barangays with the highest incidence of confirmed animal rabies cases, followed by dog bite cases admitted to the hospital from the barangays. The sample size for each KAP barangay was 200 households.

**Figure 2:**
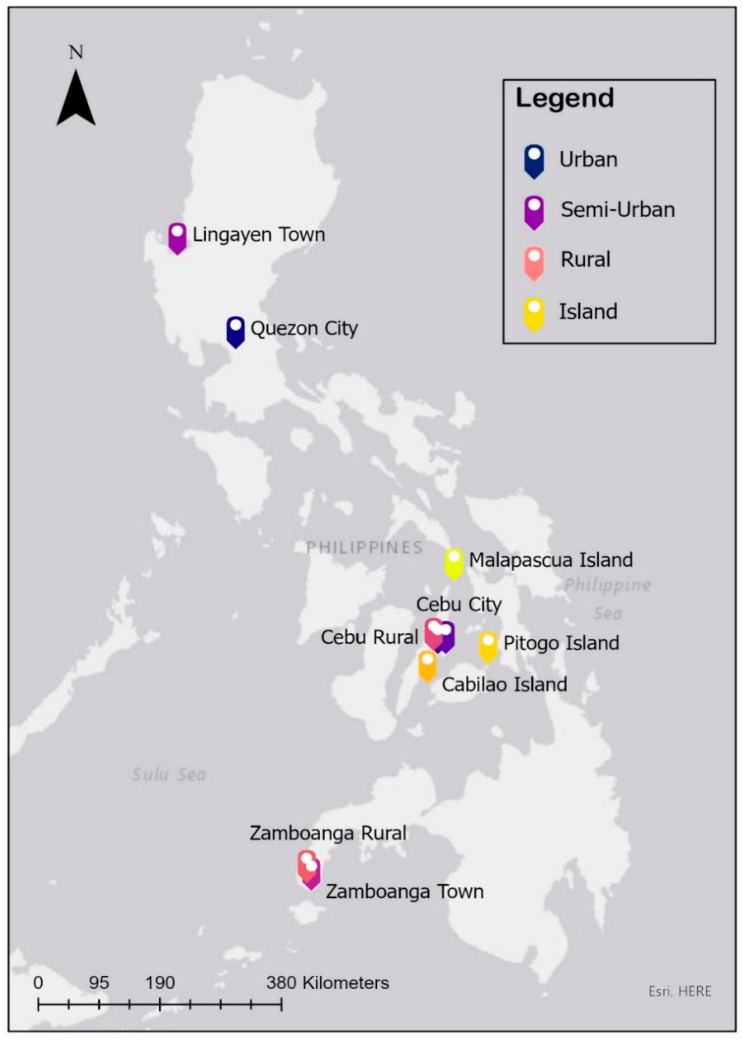
Arc GIS map of household survey locations in the Philippines; colour coded for four different categories.

Across the Philippines, several locations were surveyed (Figure 2) using the same methodology. The sample size was based on the urbanisation of the location and resources available for the survey. 

### 2.3. Ethics

The data collection was launched to generate baseline data for the anti-rabies vaccination programs in the Philippines. Verbal consent was collected from each survey participant. For consistency and accuracy in delivering the consent text, the surveyor read a pre-written text explaining the reason they were asked to participate, the scope of the questionnaire and confirming that no personal information identifying any individual would be collected during the interview. Participants were informed that they can withdraw their consent at any point during and after the interview and that they can skip questions they do not wish to answer. If participants did not agree to participate, the surveyor would record a “no” and the questionnaire automatically ended.

## 3. Results

### 3.1. Dog Demographics and Dog Ownership

**Figure 3:**
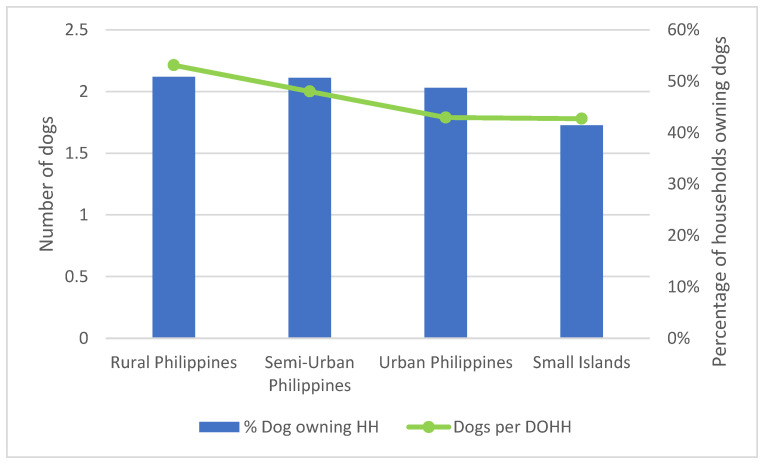
Dogs per dog owning household (DOHH) in different categories of human settlements (Rural, Semi-Urban, Urban and Islands).

Household dog ownership rates across all study areas were generally high, varying between a low of 29.2% of households owning dogs on Malapascua Island to a high of 63.16% in Lingayen town. The 10,664 households surveyed across all locations owned 9916 dogs. The average number of dogs per dog owning household (DOHH) was over one in all categories of locations (Figure 3). There are an average 0.89 dogs per household (HH) across the Philippines. There was a slight preference for male dogs, but this preference was not significant (Table 1).

In Table 1, estimated dog population for each surveyed location was calculated by extrapolating dogs per household number to total households of surveyed location. A Microsoft Access database (https://www.microsoft.com/en-ww/microsoft-365/access, accessed on 27 December 2021) generated average dogs per household for the location from number of dogs counted during the survey with reference to total households surveyed for the location. 

### 3.2. Sterilisation and Vaccination Status 

**Figure 4:**
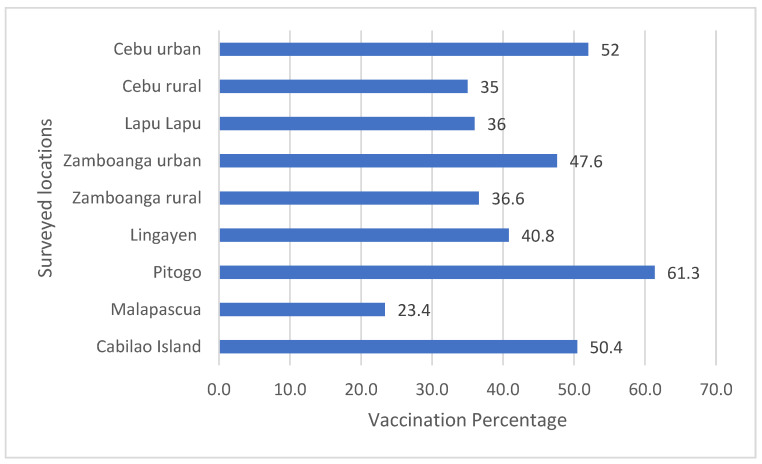
Percentages of vaccinated dogs in each surveyed location.

Vaccination levels varied between locations (Figure 4). There were no clear reasons identified why the percentage of dogs vaccinated differed.

**Figure 5:**
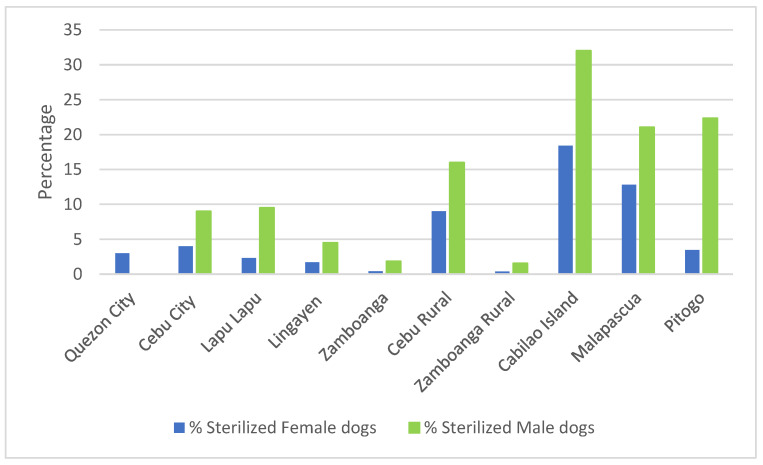
Percentage of male and female dogs sterilized by location. Note: male dogs were not recorded in Quezon City.

The percentage of dogs that were sterilized as a proportion of their sex is reported in Figure 5. Higher proportions of male dogs were sterilized compared to female dogs.

Across all locations and urbanization levels, dog owners were overwhelmingly willing to have their dogs vaccinated (Table 2). However, around a quarter to a third of owners in urban Quezon City (75.6%) and the islands Cabilao (67.9%) and Pitogo (79.4%) were unwilling to have their dogs vaccinated. The level of willingness to have dogs sterilized varied from around 10% to over 90%. It was not clear why there were such large differences in owner attitudes towards dog sterilisation from one location to another.

### 3.3. Dog Population by Human Density and Type of Human Settlement

**Figure 6:**
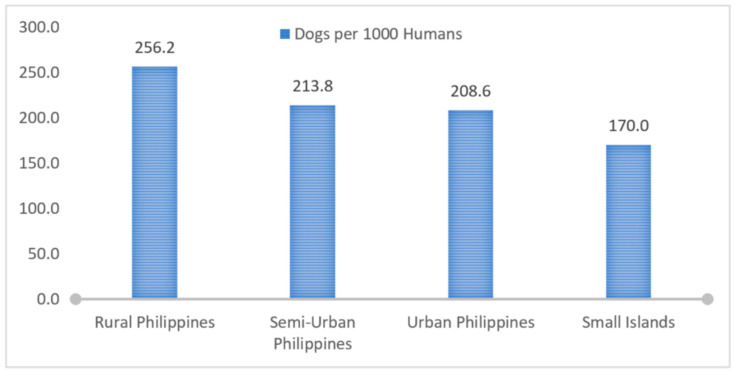
Dogs per 1000 humans based on the level of the urbanisation of the human settlement.

There was some decrease in relative dog populations as one moves from rural to urban locations, but the island locations had the lowest relative dog populations, even though human density on the islands was low to medium (Figure 6). 

Locations combined by the settlement type provide a range of dog densities per 1000 humans (Figure 6). We found 256.2 dogs (*n* = 1386, CI 95%, 249.6–262.4) per 1000 humans in rural areas, 213.8 dogs (*n* = 2354, CI 95%, 208.7–219.4) per 1000 humans in semi-urban areas, 208.6 dogs (*n* = 6224, CI 95%, 203.8–214.2) per 1000 humans in urban areas and 170 dogs (*n* = 700, CI 95%, 163.7–176.3) per 1000 humans in small islands of the Philippines. This indicates that more privately owned dogs are kept per 1000 people in rural areas compared to urban areas.

### 3.4. Dog Population and Human Density

Dog density (dogs per 1000 people) may vary with human density (humans per km^2^). Table 3 examines this relationship. Of all ten locations we surveyed, human population density data up to barangay level were only available for the three locations we presented in Table 3. We observed fewer dogs per 1000 people as human density increased in each of these three locations: Quezon City, Lingayen Town and Zamboanga rural area. 

Across our sample of 10,664 households (2086 rural and 8578 urban) we counted 2076 (95% CI: 1987–2165) and 7840 (95% CI: 7666–8014) dogs in rural and urban samples respectively. Extrapolated for the total human population and number of households present in the sample regions, as reported by the World Bank [21], we calculate an estimated average dog density of 221.1 (rural) and 209.2 (urban) dogs per 1000 people. Using the World Bank’s total human population figure (divided into rural and urban totals) across the Philippines as a whole [21], we calculate a rough estimate of 23,295,301 (95% CI, 22,515,832–24,074,770) dogs across the country, following from our rural and urban dog density estimations.

### 3.5. Level of Confinement in Different Types of Human Settlements

**Figure 7:**
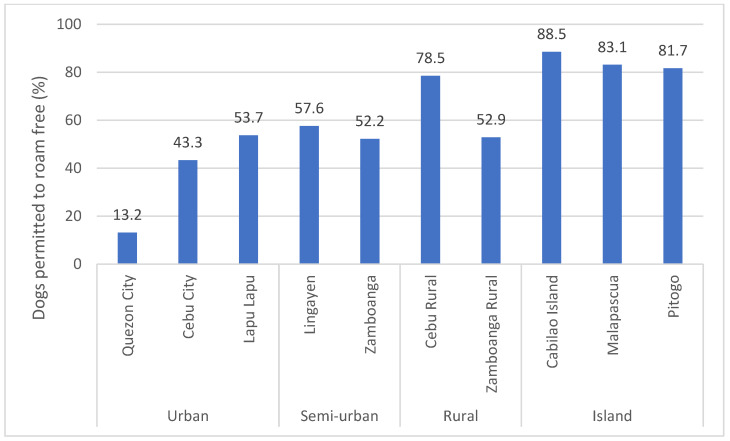
Percentage of dogs permitted to roam free in each survey location.

The level of confinement differed significantly between survey locations (X-squared = 1999, *df* = 9, *p*-Value < 0.001) and between different settlement types (urban, semi-urban, rural and island: X-squared = 1256.5, *df* = 3, *p*-Value < 0.001), appearing to increase with the degree of urbanisation (Figure 7). A chi-squared pairwise comparison (with Bonferroni *p*-Value correction) of the four grouped settlement categories reveals significant differences in confinement proportions between all settlement categories (*p* < 0.001 for all pairwise comparisons) with the exception of the semi-urban and rural category comparison (*p* = 0.0533). This supports the hypothesis of increasing confinement practices with increasing degree of urbanisation.

### 3.6. Knowledge Attitude and Practice (KAP) Survey Finding from Quezon City

#### 3.6.1. Human Demographics

In Quezon City, 1741 households were interviewed using a detailed KAP questionnaire prepared in Epicollect5. The sample consisted of 1026 (63.6%) female and 587 (36.4%) male interviewees. Of these, 938 (58.2%) came from dog owning households and 675 (41.8%) from non-dog owning households. Of the 936 dog owning households, 651 (69.6%) owned one dog, 187 (20%) owned two dogs, 61 (6.5%) owned three dogs, 18 (1.9%) owned four dogs, 11 (1.2%) owned five dogs and 14 (1.5%) owned six or more dogs. Over 90% of interviewees (865) were the main caretaker for the dog(s). The main reasons for owning a dog were Pet/Companionship (49.1%, 645) and protection of the property/crops (49.0%, 645). Another 1.8% (23) said that they owned dogs to breed, and one household (0.1%) had a dog for food/to be eaten. Most of the homes had a fenced-in yard (63.4%).

#### 3.6.2. Rabies and Dog Bites

Regarding symptoms of rabies in dogs, 96.4% (1552) reported that they have heard about rabies. When asked “Do you think it is possible for you or your family to get rabies?” 82.7% (1332) said yes and 8.8% (142) and 8.5% (136) either said no or did not know, respectively. When asked if the interviewee knew how rabies in dogs could be prevented, 60% (1259) knew that dogs should be vaccinated annually against rabies and another 10% (201) knew that dogs should get an injection but did not know what the injection would be. A small proportion (8%, 179) thought that impounding dogs would be the best method to prevent rabies in dogs.

#### 3.6.3. Dog Demographics

Dogs were acquired in several ways (Table 4). The most common way of adding a dog to a household was by receiving it from someone (65.9%, 880) followed by being born in the household (11.8%, 158). Therefore, most owners acquired their dogs by “accident”. Less than twenty percent either adopted or bought their dog intentionally. Puppies born at the owner’s home were given mainly as gift to family and friends (43.6%) and only 15.6% were sold (Table 5). 

**Figure 8:**
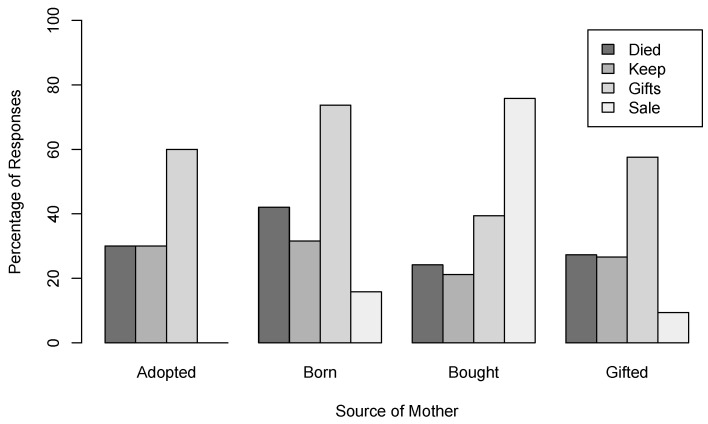
Puppy fate by mother acquisition source for Quezon City.

About one third of the 578 female dogs (33.2%, 192) in the sample had at least one litter in their life (Table 6). It is unclear, however, how many puppies survived from each litter and grew into adult dogs. Puppies that were born to bought female dogs appear to be more likely to be also sold compared to puppies born to other female dogs (Figure 8).

### 3.7. Application to the National Rabies Vaccination Program

**Figure 9:**
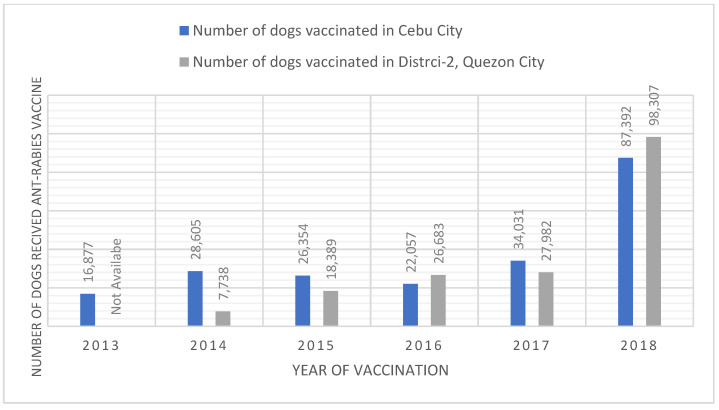
Number of dogs vaccinated between 2013 to 2018 in Cebu City and Quezon City.

After the survey results were shared internally with the local government veterinary offices, a joint vaccination program was initiated in Cebu City and District-2 of Quezon City in 2018 in coordination with the Bureau of Animal Industry and DARFO-7. In earlier rabies vaccination programs (2013–2017), an average of 25,585 and 20,198 dogs were vaccinated annually in Cebu City and Quezon City, respectively. In 2018, the vaccination program used the survey data to plan and resource (with ample vaccination doses) a new rabies vaccination drive (Figure 9). In Cebu City, the new vaccination drive lasted from February 2018 to end of August 2018; whereas in Quezon City the vaccination drive started later towards the beginning of August 2018 and continued until the first week of December 2018. As a result, numbers of rabies cases reported during 2018 were high since there was no vaccination effort during the first half of the year, yet fewer numbers of cases observed in both places during 2019 indicate the efficacy of the program.

**Figure 10:**
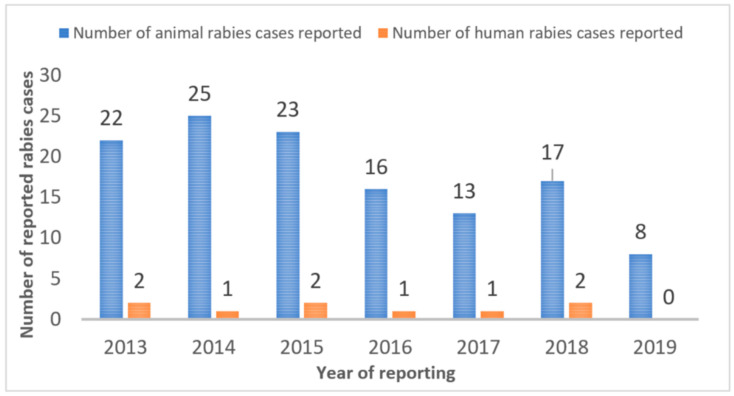
Reported rabies cases in animals and humans in Quezon City, pre- and post-2018 vaccination drive.

**Figure 11:**
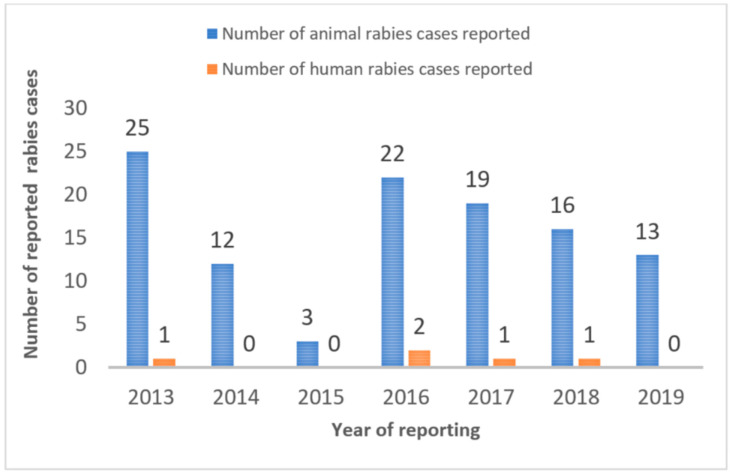
Reported rabies cases in animals and humans in Cebu City, pre- and post-2018 vaccination drive.

In both cities, the rabies incidence in animals and humans has declined (Figure 10 and Figure 11). For 2019, the cities reported zero cases of rabies in humans. If rabies vaccination programs continue to reach the 2018 numbers, rabies cases should continue to decrease in animals and human rabies cases should remain at zero.

## 4. Discussion

Our household surveys across all the locations found that 50% of households own one or more dogs. This was independent of the location’s urbanisation status (rural, semi-urban, or urban). Only on small, isolated islands did we find a lower ownership rate of approximately 40% (Table 1). This suggests that the size of the owned dog population in an area of the Philippines is dependent on the number of dogs owned per dog owning household. This statistic differed by urbanization status: highest in rural areas (2.21 dogs/DOHH) and lowest in urban areas (1.73 dogs/DOHH). Dog owners were found to possess a marginal bias to owning male dogs in preference to female. Confinement practices varied dramatically with level of urbanisation, with the percentage of dog owners willing to allow their dogs to roam the street increasing with decreasing urbanization: 29.3% in urban areas, 54.7% in semi-urban, 60% in rural and 84.5% on the small islands. Often these free roaming owned dogs are mistaken for un-owned street dogs during the planning of a large-scale mass vaccination programs and targeted for catching. However, door to door vaccination programs may simultaneously provide a simpler and more cost-effective means to vaccinate such a population and help educate owners concerning the necessity of annual vaccination. Dog owning data also have the potential to inform projected vaccination goals in planning large scale mass vaccination programs, using dog to household ratios (Table 1) combined with the total household numbers available from human census data.

We have also presented data in the form of dogs per 1000 humans for all types of settlements, which may be extrapolated to any area of the Philippines. Further, based on the assumption of sample representativeness to the country as a whole, we used urban and rural dogs per 1000 humans to estimate the owned dog population of the Philippines country. Such total country estimates are necessary for central government agency plans for large scale vaccination programs, especially with respect to securing sufficient vaccine numbers. While more data from other parts of the Philippines are needed to validate our estimate, in the meantime, it serves as a guide for other Asian and African countries to establish similar ratios for each region and, thus, plan effective large scale vaccination programs.

The sterilization rate was found to be very low in all surveyed locations; a comparatively higher sterilisation rate was found in male dogs. Owners’ willingness for male dog sterilisation was also found to be higher suggesting that dog owners prefer intact female dogs with the potential to breed. In Quezon City, when we used the longer version of our questionnaire, we found that the gifting of dogs is a common cultural practice: 65.9% of dogs were acquired as gifts (Table 4) and upon asking about the fate of newborn puppies, 43.6% of dog owners gave them away as gifts (Table 5). On the other hand, we found a high willingness for vaccinating owned dogs against rabies in all the surveyed locations. This is encouraging for the prospect of potential large scale rabies vaccination programs.

In Quezon City, we found that people are keeping dogs either as a companion (49%) or as a guard (49%), with the majority (69.6%) keeping only a single dog. Almost all the interviewed households (96.4%) knew about rabies disease, but only 60% of them knew about the necessity of annual re-vaccination. This shows that there is broad scope for and benefit in an awareness and education program alongside the annual vaccination program.

There has been a shift in rabies and humane dog population management in the past decade. Successful programs now demand more evidence of impact beyond just the number of vaccine doses administered and the number of reported rabies cases in animals [22]. There are long-standing and successful anti-rabies programs in other parts of the world that did not start by determining dog populations. However, in the Philippines, South Asia and Africa, the persistence of rabies begs for a re-evaluation of the approaches used to eliminate the virus.

Despite the lingering rabies caseload in the Philippines, the government is committed to eradicating the virus with mass-dog-vaccination programs. The locations in this study were chosen specifically because they were places where local veterinary offices decided their programs needed to be more evidence-based and sought support from Humane Society International. The results of the surveys in this report indicate that there are better approaches to estimating the dog population than the 1 dog per 10 people rule commonly used by municipalities in the Philippines.

This study shows that standard estimation of the dog population (using 1 dog per 10 people) undercounts local dog populations by 2–3-fold. As a result, dog vaccination programs carried out in the past by Philippines municipalities never achieved a 70% coverage of dogs in the community. A revised human-dog ratio to estimate local dog populations would help secure higher (and more effective) coverage of rabies vaccination. A single parenteral dog rabies-mass vaccination campaign achieving coverage of at least 70% appears to be sufficient to interrupt rabies transmission to humans for up to 6 years [23].

In addition to evidence-based planning, Quezon City and Cebu City used a specifically designed mobile phone application for household surveys and to track their vaccination program. The increased data accuracy and simplified data compilation provide additional information that can be used for monitoring and evaluation purposes.

## 5. Conclusions

Dogs are closely associated with humans across the world. Based on this study, we conclude that using a dog per 1000 humans metric would be a helpful approach when determining the needs of community rabies vaccination programs. The metric is easy to understand and easy to use. Furthermore, a breakdown into dog—human ratio for rural, semi-urban, urban and small islands has potential to aid the planning of mass vaccination and dog sterilisation programs across the Philippines. The existence of human rabies cases in Quezon City despite people having knowledge of rabies disease and knowing the risk of rabies transmission demand a better rabies vaccination approach. Implementing the baseline dog surveys in Cebu City and Quezon City ultimately led to plans for a broader vaccination campaign in both urban centers and, due to a correction in the number of vaccines doses needed, a 3- to 4-fold increase in dog vaccination coverage. Nationally, such a survey-driven vaccination program could improve rabies elimination efforts and result in a greater likelihood that the Philippines could achieve rabies-free status by 2030.

## Figures and Tables

**Table 1:** Number of households surveyed, dog population estimates, dogs per 1000 humans, percentage of dog owning households, average number of dogs per household, dogs per dog owning household, percentage of female dogs in the population and percentage of dogs allowed to roam freely for some or all of the day in different locations [DOHH—Dog-owning Household; HH—Household].

Area/City Name	Human Density (Per km^2^)	HH Sample	Total Dogs (Estimated)	Dogs per 1000 Human	% DOHH	Dog per HH	Dogs per DOHH	% Female Dogs	% Dogs Unconfined
Quezon City	18,223	3500	648,747	220.95	54.91	0.95	1.71	44.2	15
Cebu City	13,696	1603	135,548	176.80	46.10	0.82	1.78	46.6	43.3
Lapu Lapu City	6331	1121	73,146	179.20	43.26	0.81	1.76	43.8	53.7
Urban Philippines	14,601	6224	857,441	208.57	50.55	0.91	1.73	44.5	29.3
Lingayen town	1560	1113	29,377	284.40	63.16	1.22	1.90	48.1	57.6
Zamboanga town	2321	1241	86,787	235.60	39.40	0.85	2.20	42.6	52.2
Semi Urban Philippines	2124	2354	116,164	213.76	50.64	0.92	2.00	44.0	54.7
Cebu Rural	662	386	31,715	203.53	57.0	1.05	1.84	39.5	78.5
Zamboanga Rural	888	1000	116,246	275.70	48.50	1.19	2.40	41.6	52.85
Rural Philippines	813	1386	147,961	256.23	50.87	1.15	2.21	41.2	60.0
Cabilao Island	577	190	676	152.75	36.3	0.59	1.64	33.6	88.5
Malapascua Island	3266	130	854	166.56	29.2	0.59	2.03	50.6	83.1
Pitogo Island	426	380	4066	174.07	48.2	0.86	1.78	43.5	81.7
Three Islands	514	700	5595	170.03	41.4	0.76	1.78	42.4	84.5

**Table 2:** Willingness of dog owners to vaccinate and sterilize their pet dogs.

City/Town/Area Name	Anti-Rabies Vaccination Status (% Coverage)	% Owner Willing to Vaccinate Their Dog	% Owner Willing to Sterilize Their Female Dogs	% Owner Willing to Sterilize Their Male Dogs
Urban Philippines				
Quezon City	73.4	75.6	10.4	12.8
Cebu City	52	96.8	40.6	48.1
Lapu Lapu	36.0	96.8	59.0	65.9
Semi Urban Philippines				
Lingayen	40.8	99.1	18.4	31.5
Zamboanga	47.6	98.7	31.3	34.6
Rural Philippines				
Cebu Rural	35	91.5	63.2	60.7
Zamboanga Rural	36.6	96	6.6	12.8
Islands				
Cabilao	50.4	67.9	64.5	86.3
Malapascua	23.4	100	94.1	93.3
Pitogo	61.3	79.4	71.4	74.7

**Table 3:** Dogs per 1000 humans based on the density of the human population in Quezon City, Lingayen town and Zamboanga rural Each location was divided into three density categories (low, medium and high).

Human Density per km^2^	Dogs per 1000 Humans	Density Level for the Location
**Quezon City**		
Below 20,000	234	Low
20,001 to 50,000	223	Medium
Above 50,001	175	High
**Lingayen Town**		
Below 1000	323	Low
1001 to 3000	320	Medium
Above 3001	242	High
**Zamboanga Rural**		
Below 1000	302	Low
1001 to 10,000	282	Medium
Above 10,000	242	High

**Table 4:** Number and percentage of dog’s acquisition by pet owners in Quezon City.

Where Did You Get This Dog from?	Number	Percentage	Source Category	Number	Percentage
Adopted from the street outside this Barangay	6	0.4	Adopted	53	4.0
Adopted from the street in this Barangay	32	2.4			
Adopted from a shelter	1	0.1			
Adopted from another person outside this Barangay	6	0.4			
Adopted from another person in this Barangay	8	0.6			
Born in this household	158	11.8	Born to household	158	11.8
Bought by the owner/household outside this Barangay	137	10.3	Bought	230	17.2
Bought by the owner/household within this Barangay	93	7.0			
Given to the owner/household	880	65.9	Gifted	880	65.9
Other	14	1.0	Other	14	1.0
TOTAL	1335	-			

**Table 5:** Practices followed by pet owners for puppies born at their home in Quezon City.

What of the Following Do You Usually Do with Puppies? Note That Multiple Responses Can Be Given per Respondent	Number	Percentage	Fate Category	Number	Percentage
Give them away as gifts to friends/family	115	43.6	Gifts	115	43.6
Keep puppies in my home	51	19.3	Keep	51	19.3
Puppies died	57	21.6	Died	57	21.6
Sell to other dog owners outside my Barangay	20	7.6	Sale	41	15.6
Sell to other dog owners in my Barangay	21	8.0			
TOTAL	264	-	-	-	-

**Table 6:** If a female dog had litter in her life based on the source of acquisition in Quezon City.

Source	Has She Ever Had Puppies?		
	Yes	No	% Yes
Adopted	10	11	47.6
Born to household	20	46	30.3
Bought	32	84	27.6
Gifted	129	237	35.2
Other	1	2	-
TOTAL	192	380	33.6

## Data Availability

All the data collected during the study are stored in Microsoft Access Database in raw form and in Microsoft Excel in calculated form with the first author (Amit Chaudhari) and those can be shared in calculated or raw forms if needed for validation. Data collected from Quezon City through Epicollect5 are also stored on a Epicollect5 online project account.
